# Screening the Expression of ABCB6 in Erythrocytes Reveals an Unexpectedly High Frequency of Lan Mutations in Healthy Individuals

**DOI:** 10.1371/journal.pone.0111590

**Published:** 2014-10-31

**Authors:** Magdalena Koszarska, Nora Kucsma, Katalin Kiss, Gyorgy Varady, Melinda Gera, Geza Antalffy, Hajnalka Andrikovics, Attila Tordai, Maciej Studzian, Dominik Strapagiel, Lukasz Pulaski, Yoshihiko Tani, Balazs Sarkadi, Gergely Szakacs

**Affiliations:** 1 Hungarian National Blood Transfusion Service, Budapest, Hungary; 2 Institute of Enzymology, Research Centre for Natural Sciences, Hungarian Academy of Sciences, Budapest, Hungary; 3 Molecular Biophysics Research Group of the Hungarian Academy of Sciences, Semmelweis University, Budapest, Hungary; 4 Department of Molecular Biophysics, University of Lodz, Lodz, Poland; 5 Biobank Lab, Department of Molecular Biophysics, University of Lodz, Lodz, Poland; 6 Japanese Red Cross Kinki Block Blood Center, Osaka, Japan; Niels Bohr Institute, Denmark

## Abstract

Lan is a high-incidence blood group antigen expressed in more than 99.9% of the population. Identification of the human ABC transporter ABCB6 as the molecular basis of Lan has opened the way for studies assessing the relation of ABCB6 function and expression to health and disease. To date, 34 ABCB6 sequence variants have been described in association with reduced ABCB6 expression based on the genotyping of stored blood showing weak or no reactivity with anti-Lan antibodies. In the present study we examined the red blood cell (RBC) surface expression of ABCB6 by quantitative flow cytometry in a cohort of 47 healthy individuals. Sequencing of the entire coding region of the *ABCB6* gene in low RBC ABCB6 expressors identified a new allele (IVS9+1G>A, affecting a putative splice site at the boundary of exon 9) and two nonsynonymous SNPs listed in the SNP database (R192Q (rs150221689) and G588 S (rs145526996)). The R192Q mutation showed co-segregation with reduced RBC ABCB6 expression in a family, and we found the G588 S mutation in a compound heterozygous individual with undetectable ABCB6 expression, suggesting that both mutations result in weak or no expression of ABCB6 on RBCs. Analysis of the intracellular expression pattern in HeLa cells by confocal microscopy indicated that these mutations do not compromise overall expression or the endolysosomal localization of ABCB6. Genotyping of two large cohorts, containing 235 and 1039 unrelated volunteers, confirmed the high allele frequency of Lan-mutations. Our results suggest that genetic variants linked to lower or absent cell surface expression of ABCB6/Langereis may be more common than previously thought.

## Introduction

ATP-binding cassette (ABC) transporters make up one of the largest protein families from bacteria to man [1]. ABC proteins are essential for many physiological processes, and mutations in these genes have been linked to several human genetic disorders including cystic fibrosis, connective tissue mineralization, neurological disease, retinal degeneration, cholesterol and bile transport defects, anemia and gout [2]. Human ABC transporters transport a large variety of endogenous substrates and xenobiotic compounds. Despite extensive studies, the physiological function of several ABC transporters remains unknown.

ABC proteins are divided into seven subfamilies, based on domain organization and sequence homology. ABCB6 belongs to the ABCB subfamily, which includes P-glycoprotein (ABCB1/MDR1) causing multidrug resistance of cancer cells [3]; the TAP1/2 complex (ABCB2-3) mediating the translocation of immunogenic peptides from the cytosol into the endoplasmic reticulum [4]; ABCB4-MDR3 influencing the translocation of phosphatidylcholine [5], and ABCB11 involved in the secretion of bile acids [6].

ABCB6 is one of the four ABC transporters that were assigned to the mitochondria. Initially, ABCB6 was reported to be a regulator of de novo porphyrin biosynthesis by promoting the ATP-dependent mitochondrial uptake of porphyrins [7]. Although initial findings suggested that loss of one Abcb6 allele in embryonic stem (ES) cells impairs porphyrin synthesis, mice derived from these stem cells were phenotypically normal [8]. Recent studies have challenged the paradigm linking the function of ABCB6 to mitochondria, and suggested that ABCB6 is localized in the endolysosomal continuum including the plasma membrane [9], the Golgi apparatus [10], and organelles of the vesicular system [11], [12], [13], [14]. ABCB6 is upregulated during erythroid differentiation, and although a proportion is lost during reticulocyte maturation through secreted exosomes, ABCB6 is expressed in mature red blood cells at relatively high levels [15]. In 2012, ABCB6 was revealed as the molecular basis of the Lan blood group antigen [16]. Lan is a high frequency blood group, expressed in more than 99.9% of most populations. Consequently, Lan-negativity is extremely rare, estimated to appear in approximately 0.005% of the Caucasian population [17]. Consistent with the knock-out mouse study, Lan-negative individuals lack an overt phenotype, arguing against a vital role of ABCB6 in porphyrin synthesis. Still, the Lan antigen is medically relevant since individuals with Lan(−) blood group may develop anti-Lan antibodies, which can cause transfusion reactions or hemolytic disease of the fetus or newborn.

The NCBI SNP database lists 417 ABCB6 variants/SNPs, of which 34 have been associated with the Lan-negative phenotype. All the Lan-negative individuals genotyped so far have inherited two recessive null mutations. Heterozygous mutations result in reduced erythrocytic protein levels, indicating bi-allelic expression of ABCB6. Based on the Phase 1 publication of the 1000 Genome genotype data [18], the global minor allele frequency (MAF) of the Lan-associated SNPs is low, indicating that individual variations are relatively rare. Recent studies have associated mutations in ABCB6 with various hereditary diseases including ocular coloboma [19], dominant familial pseudohyperkalemia [20] and dyschromatosis universalis hereditaria (DUH) [21]. The pathophysiological role of ABCB6 in these conditions is not known since it is not possible to directly correlate sequence variations to ABCB6 function or expression. This is partly due to the lack of our understanding of the physiological function and the structure-activity relationships within ABCB6, and also to the complex regulation of protein synthesis, maturation, trafficking and posttranslational modifications of membrane proteins.

Recently, we have characterized the quantitative expression of the ABCG2 multidrug transporter corresponding to the Jr^a^ blood group in human erythrocytes and have shown that pharmacologically relevant polymorphisms are associated with significantly reduced ABCG2 protein expression [22]. Here we show that RBC ABCB6 expression levels display a large variation in healthy individuals, with an unexpectedly high frequency of low expressors. Genotype screening of two large cohorts (containing 235 and 1039 unrelated volunteers) confirmed that genetic variations underlying low surface ABCB6 expression are more common than previously inferred based on the frequency of Lan-negative blood type.

## Methods

### Probands

The “discovery cohort” consisted of 47 healthy, Hungarian unrelated individuals [(12 males and 35 females; median age: 37 (26–66)]. Family members (n = 4) of 2 healthy volunteers with low ABCB6 expression and two Lan-negative (Lan−) individuals with a previous history of hemolytic disease of the newborn caused by anti-Lan antibody were investigated additionally [23]. The Hungarian genetic screening cohort consisted of 184 healthy, unrelated first or second time Hungarian blood donors ((94 males, 90 females; median age 37 (22–76)). The Polish genetic screening cohort included 1039 healthy, unrelated subjects (555 males, 484 females, median age 39 (19–76)). This sample was randomly selected within the “normal Polish population” genetic collection at the Biobank, Department of Molecular Biophysics, University of Lodz. Genetic material for this collection was sampled in 2011–2012 within the EU-funded TESTOPLEK research project. A professional public opinion polling and survey-taking company (SMG/KRC Poland, a Millward Brown subsidiary) was subcontracted to select a cross-sectional sample of >10000 individuals from the population at large, with representation from each geographical region of Poland (number of samples from each powiat/county proportional to the population of the county). The individuals were selected from public records, visited in their domiciles by SMG/KRC pollsters and asked to provide genetic material (saliva, see below) and to fill out a general anthropological and medical questionnaire. Since proband selection was random from the overall population, the probands were not necessarily healthy individuals, although the sample excluded hospitalized patients and those unable to give informed consent. All subjects gave their written informed consent to participate in the study. This study was approved by the respective regional ethical committees (The University Committee on Bioethics Research (University of Lodz); Scientific and Research Ethics Committee of the Healthcare Scientific Council of Hungary), and all procedures were performed in accordance with the Declaration of Helsinki.

One patient’s sample was obtained through the NIH Undiagnosed Diseases Program (UDP) in the United States [24]. The patient was enrolled in clinical protocol 76-HG-0238, approved by the Institutional Review Board of the National Human Genome Research Institute. The parents gave written, informed consent.

### Genotyping

In the Hungarian cohort, genomic DNA was isolated from EDTA anticoagulated peripheral blood by Gentra Purege Blood Kit (Qiagen, Hamburg, Germany). Sanger sequencing of the ABCB6 coding region and exon-intron boundaries (exons 1–19, NCBI Reference Sequence: NT_005403.18) was performed by Applied Biosystems 310 Genetic Analyzer (Life Technologies, Carlsbad, USA). Screening for ABCB6 variants at R192 (rs150221689) or G588 (rs145526996) was performed by the PCR restriction fragment length polymorphism method using SsiI and Sau96I, respectively. Digested PCR products were size-separated by electrophoresis on 2% agarose gels and visualized by ethidium bromide staining. For the detection of ABCB6 R192 codon variants the primer pair for amplification of exons 1–2 (ABCB6-2 and ABCB6-4) was used according to Helias et al. [16], while for the detection of ABCB6 G588 S variants the following primers were used ABCB6-37 [16] and ABCB6-13R (5′- GGAGGCTGCACCATTCAATC-3′).

For the Polish population study, saliva was collected into Oragene OG-500 DNA collection/storage receptacles (DNA Genotek, Kanata, Canada) and genomic DNA was subsequently isolated by the MagNA Pure LC DNA Isolation Kit - Large Volume (Roche, Basel, Switzerland) with its final concentration normalized to 0.2 µg/ml. Screening for expected ABCB6 SNP variants at R192, G588, R276 as well as the exon 9 boundary splice site was performed by High Resolution Melting (HRM) analysis of PCR products amplified and subsequently melted on a BioRad C1000/CFX384 thermocycler. The reaction used 500 pg genomic DNA as template, 2xGoTaq polymerase master mix (Promega, Madison, USA) and LCGreen dye (BioFire Defence, Salt Lake City, USA). HRM curves were interpreted using the Precision Melt Analysis Software from BioRad. Samples with divergent melting curves were selected for re-amplification, cloning and Sanger sequencing to confirm the presence of respective SNPs.

The following primers were used for HRM analysis (all at 59°C annealing temperature):

ABCB6-HRM-R192-F 5′-GTTCAGTTTAGCCTGTGGGTG-3′
ABCB6-HRM-R192-R 5′-CAAACAGCCCTCCAGAGACC-3′
ABCB6-HRM-R276-F 5′-CTGGGGCTCATGGGTTTG-3′
ABCB6-HRM-R276-R 5′-GGCACCAACACATTGAGTG-3′
ABCB6-HRM-G588-F 5′-TGGAGCAGGGCCCCTTC-3′
ABCB6-HRM-G588-R 5′-CATCGGCATAGCTGAAGTGC-3′
ABCB6-HRM-IVS9-F 5′-GCGCATACTTTGTCACTGAGCAG-3′
ABCB6-HRM-IVS9-R 5′-CCCACTCTCTCATCCAAGATCC-3′

### Quantitative analysis of ABCB6 expression by flow cytometry

Freshly drawn human blood (25 µl) was diluted in 4 ml of phosphate buffered saline (PBS), the cells were centrifuged at 3,000×g for 10 min. Samples were processed within 3 hours, or stored for a maximum of 2 days after fixation in 1% PFA for 60 min at 25°C. Antibody staining was performed for 40 min at 37°C by using the Human monoclonal anti-Lan (Clone OSK43; IgG1κ) (0.7 µl/tubes) specifically recognizing ABCB6 protein [16], or without primary antibody (negative staining). Antibody concentrations for ABCB6 labeling in red cells were carefully calibrated to provide maximum labeling in a full range of protein expression. After washing out the primary antibodies, secondary antibodies corresponding to the IgG type and labeled with phycoerythrin (PE) were added to the cells (Goat F(ab’)2 fragment anti-human IgG-PE (Beckman Coulter, PNI 1626)), in 50× dilution, incubated for 30 min at 37oC, washed and resuspended in PBS. The labeled samples were subjected to flow cytometry (FACS); intact red cells were gated based on the forward scatter (FSC) and side scatter (SSC) parameters. Intact cells were analyzed for antibody staining by a FACSCalibur flow cytometer (excitation wavelength: 488 nm (Argon ion laser) emission filters: 585/42 nm for PE). RBC ABCB6 was measured 4 times in each individual (Figure S1); relative ABCB6 expression was calculated by dividing the median values obtained with the primary and secondary antibodies with the weighted average of the median values obtained with secondary antibody only. The weighted average (WA) was calculated as follows: WA = (average secondary fluorescence of all measured samples*2+individually measured secondary fluorescence)/3. Surface expression of ABCB6 variants expressed in K562 cells was performed as described previously [15].

### Mutagenesis

cDNA encoding wild-type human ABCB6 (NM_005689) bearing an HA-tag at its C-terminus [9], [15] was mutated using the QuikChange site-directed mutagenesis kit (Stratagene) using the following primers:

ABCB6-R192W-F 5′-CCTGTGGGTGCTGtGGTATGTGGTCTCTGG-3′
ABCB6-R192W-R 5′-CCAGAGACCACATACCaCAGCACCCACAGG-3′
ABCB6-R192Q-F 5′-CCTGTGGGTGCTGCaGTATGTGGTCTCTGG-3′
ABCB6-R192Q-R 5′-CCAGAGACCACATACtGCAGCACCCACAGG-3′
ABCB6-G588S-F 5′-CCCCTTCGCTTTCAGAAGaGCCGTATTGAGTTTGAG-3′
ABCB6-G588S-R 5′-CTCAAACTCAATACGGCtCTTCTGAAAGCGAAGGGG-3′

Mutations were confirmed by sequencing.

### Cell lines

The HA-tagged ABCB6 and its variants (ABCB6-G588S-HA, ABCB6-R192Q-HA or ABCB6-R192W-HA, respectively) were cloned into a pSEW lentiviral vector and stably expressed in K562 and HeLa cells by lentiviral transduction. Lentiviral particles were produced in HEK293T cells transfected by the calcium phosphate co-precipitation method according to manufacturer’s protocol [25]. Viral titers of the supernatants were estimated by flow cytometric measurement of HEK cells infected with a dilution series of the supernatants; transduced cells were selected with 2 ug/ml puromycin for 2 weeks.

The K562 cell line (ATCC) was grown in RPMI 1640 medium without nucleosides, supplemented with 10% (v/v) fetal bovine serum (Invitrogen Cat.No. 10106-169) and with 2 mM glutamine, 100 units/mL penicillin, and 100 units/mL streptomycin (Invitrogen-Gibco, Carlsbad, CA, USA) at 37°C in humidified air/CO2 (19∶1) atmosphere. The HeLa cell line (ATCC) was grown in DMEM, supplemented with 10% (v/v) fetal bovine serum (Invitrogen Cat.No. 10106-169) and with 2 mM glutamine, 100 units/mL penicillin, and 100 units/mL streptomycin (Invitrogen-Gibco, Carlsbad, CA, USA) at 37°C in humidified air/CO2 (19∶1) atmosphere. Cell lines were regularly screened with the Mycoplasm Detection Kit (Lonza), and the assays were carried out in mycoplasma-negative cells.

### Confocal Microscopy

Cells at 70%–80% confluency were gently washed with Dulbecco’s modified phosphate-buffered saline (DPBS), fixed with 4% paraformaldehyde (PFA) in DPBS for 10 minutes at room temperature, and permeabilized in methanol for 90 seconds at room temperature. The samples were then blocked for 1 h at room temperature in DPBS containing 2 mg/mL BSA (Sigma, A7030), 1% fish gelatin from cold water fish skin (Sigma, G7765), 0.1% Triton-X 100 and 5% goat serum (Sigma, G9023). After blocking, the cells were incubated for 1 h at room temperature with the primary antibody diluted in blocking buffer. After washing with DPBS, the cells were incubated for 1 hour at room temperature with the respective Alexa Fluor-conjugated secondary antibody diluted at 1∶250 in blocking buffer. Where indicated, DAPI was diluted in DPBS and added to the cells after the incubation with the secondary antibody, for 10 minutes at room temperature. Wheat Germ Agglutinin and LysoTracker were added to living cells before the immunostaining procedure and incubated for 10 min or 30 min at 37C, respectively. To label cellular organelles, the following dyes and antibodies were used: LysoTracker Red DND-99 (Invitrogen, L7528), DAPI (49,6-diamidino-2-phenylindole, dihydrochloride, Sigma D9564), Alexa Fluor 633 conjugate of WGA (Invitrogen, W21404), anti-COX IV (3E11) Rabbit mAb #4850 (Cellular Localization IF Antibody Sampler Kit, Cell Signaling, #4753), anti-Calnexin (Rabbit, Cell Signaling, #4753), anti-ABCB6-567 [9], anti-HA. As secondary antibodies we used Alexa Fluor 488 Goat Anti-Mouse IgG (H+L) (Invitrogen, A11001), Alexa Fluor 594 Goat Anti-Rabbit IgG (H+L) (Invitrogen, A11012) and Alexa Fluor 594 Goat Anti-Mouse IgG (H+L) (Invitrogen, A11005). As isotype controls reagent grade IgG from mouse and rabbit serum were used (Sigma). Samples were studied with an Olympus IX-81/FV500 laser scanning confocal microscope, using an Olympus PLAPO 606 (1.4 NA) oil immersion objective (Olympus Europa GmbH). Dual channel colocalization analysis was performed by the ImageJ software with the Colocalization Threshold and Colocalization Test plugins. Averaged Pearson’s coefficients are reported for each subcellular marker from 5 random pictures [26], [27].

## Results and Discussion

**Figure 1:**
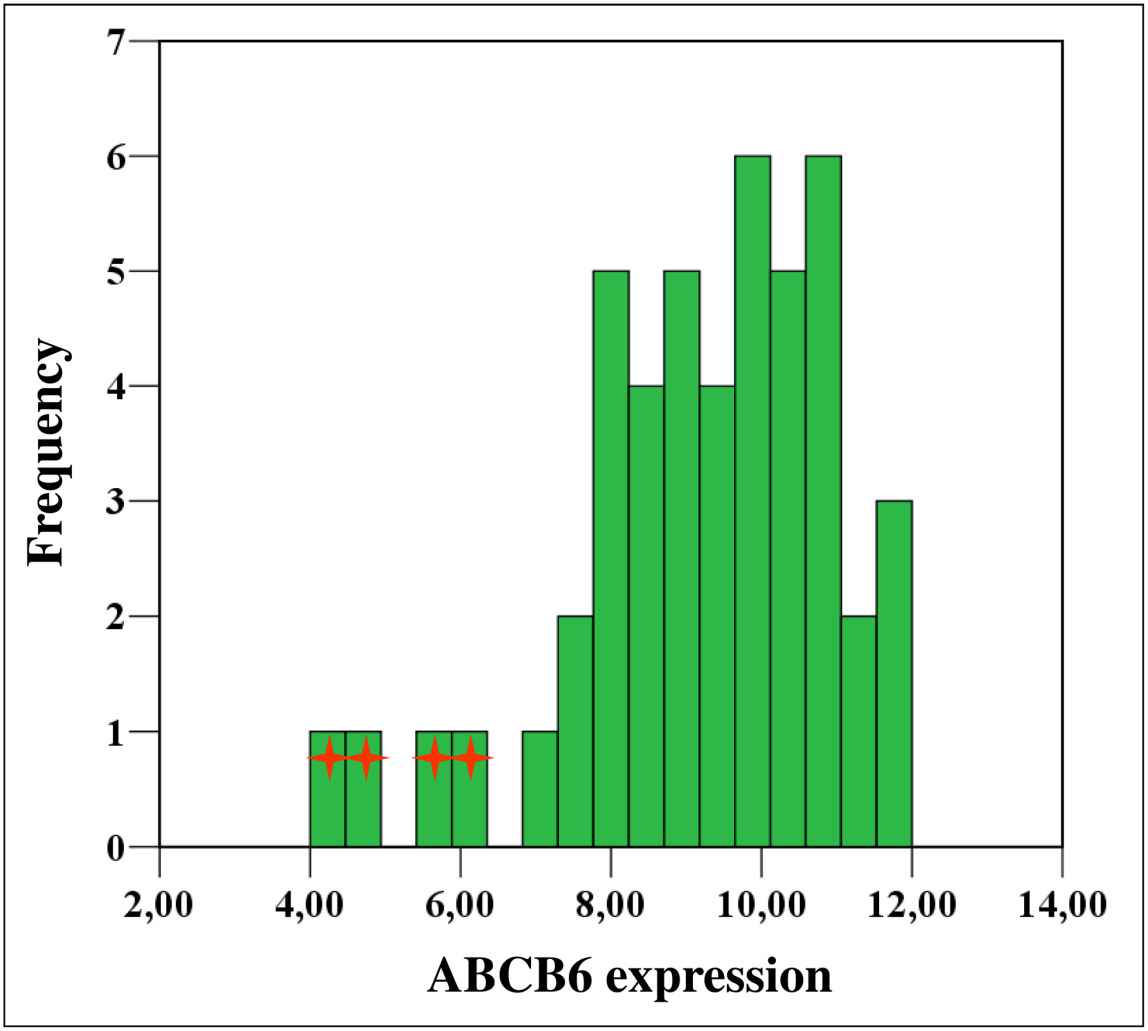
Erythrocytic ABCB6 expression in 47 healthy individuals. Histogram illustrating the distribution of erythrocytic ABCB6 expression in 47 unrelated healthy individuals, as measured by a quantitative FACS assay using the OSK43 monoclonal antibody specifically recognizing a surface epitope of ABCB6 (see Methods). The four individuals exhibiting significantly lower ABCB6 levels (below the 10^th^ percentile) are labeled by asterisks.

Population analysis of gene expression is typically achieved by quantifying levels of mRNA; however, gene expression is also a function of protein translation and turnover [28]. We used a flow cytometry-based assay for the quantitative determination of red blood cell ABCB6 expression in 47 unrelated, healthy individuals. ABCB6 expression was measured with the OSK43 monoclonal antibody specifically recognizing the Lan blood group antigen that is carried by ABCB6 [16]. Significant ABCB6 levels, encompassing a wide range of expression were detected in all but four individuals, who showed significantly lower ABCB6 levels (below the 10^th^ percentile, Figure 1, Figure S1). Differences in RBC ABCB6 expression could not be attributed to age or sex. Since mutations in the *ABCB6* gene were shown to result in the rare Lan- blood type, which is characterized by the absence of ABCB6 in red blood cells [16], we sequenced the entire coding region of the *ABCB6* gene of the four individuals exhibiting reduced RBC ABCB6 expression. Sequencing revealed alterations in each subject: two unrelated subjects were heterozygous for the missense mutation c.1762G>A, p.Gly588Ser (G588 S) encoded by the minor allele of SNP rs145526996; one subject was heterozygous for a mutation affecting the splice donor site of intron 9 (IVS9+1G>A); and a fourth subject was heterozygous for the missense mutation c.574G>A,p.Arg192Gln (R192Q) encoded by the minor allele of SNP rs150221689. The G588 S mutation was previously reported to be associated with lower RBC ABCB6 levels [28], the putative splice site mutation has not been described. Mutation of arginine to tryptophan at position 192 (R192W) was shown to be a null mutation that is responsible for the Lan-negative phenotype [28] but the presence of a glutamine at the same position has not been linked to low RBC ABCB6 expression.

**Figure 2:**
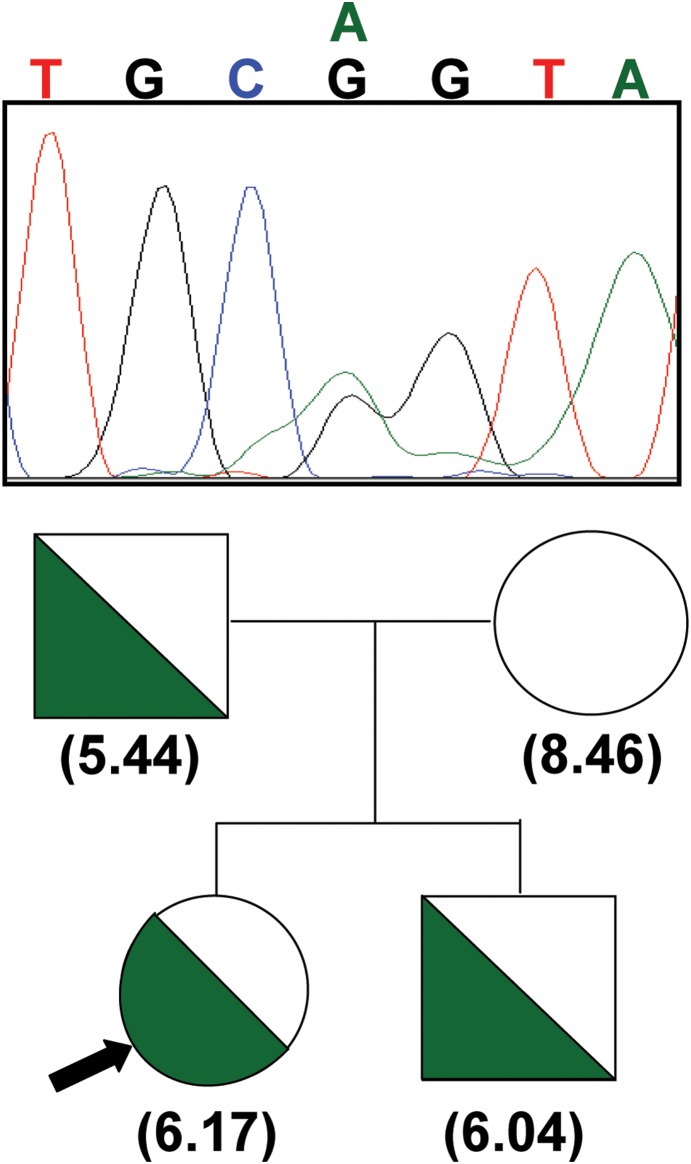
Pedigree of a family carrying the ABCB6 R192Q mutation– co-segregation of the heterozygous mutation with reduced erythrocytic ABCB6 expression levels. Blood samples obtained from the family members of the healthy volunteer proband carrying the R192Q ABCB6 mutation (indicated with black arrow) were analyzed for ABCB6 expression and the presence of the mutation. The upper panel represents a heterozygous R192Q (c.575G>A) sequence. The lower panel shows the segregation of R192Q heterozygosity (marked with green half circles and squares for females and males, respectively) with reduced ABCB6 expression in erythrocytes (values in parentheses).

In order to clarify if a direct relationship exists between the mutation R192Q and the RBC ABCB6 expression levels, we obtained blood samples from the family members of the proband carrying this mutation. We found co-segregation of the reduced RBC ABCB6 expression and the R192Q mutation, confirming the direct genotype-phenotype correlation (Figure 2). Segregation of the mutation with reduced expression indicates that the missense mutation R192Q defines a novel ABCB6 allele related to weak or no expression of ABCB6 on RBCs.

**Figure 3:**
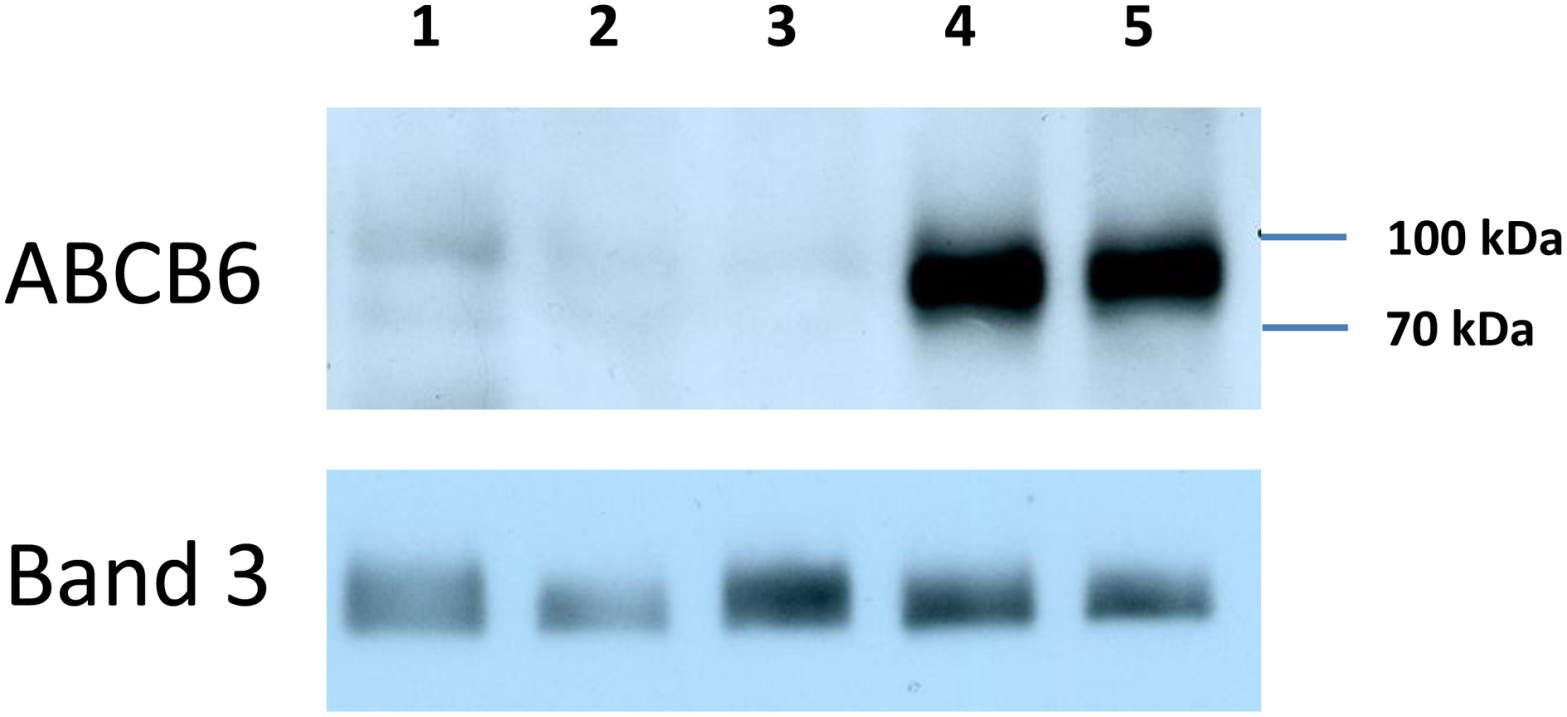
Characterization of ABCB6 mutations responsible for the Lan- blood type. RBC membrane lysates (80 ug/lane) were prepared from a compound heterozygote individual carrying the R276W and the G588S mutations (lane 1); homozygotes carrying the R192W mutation (c.574C>T, p.Arg192Trp); lanes 2, 3); or individuals carrying two wild-type alleles (lanes 4, 5). ABCB6 expression is revealed by the ABCB6-567 monoclonal antibody [9], Band III is shown as loading control.

The G588S (rs145526996) mutation was reported to result in a significant drop in RBC ABCB6 expression in heterozygous individuals in a previous study that also found decreased RBC ABCB6 levels in heterozygous individuals carrying the p.Arg276Trp mutation [28]. Our study confirms the association of both of these mutations with reduced RBC ABCB6 levels. We were unable to detect ABCB6 expression in the erythrocyte of a compound heterozygote patient referred to us carrying both the R276W and the G588S mutations (c. 826C>T, p.Arg276Trp/c.1762G>A, p.Gly588Ser), corroborating the conclusion that these mutations correspond to weak or no expression of ABCB6 on RBCs (Figure 3).

**Figure 4:**
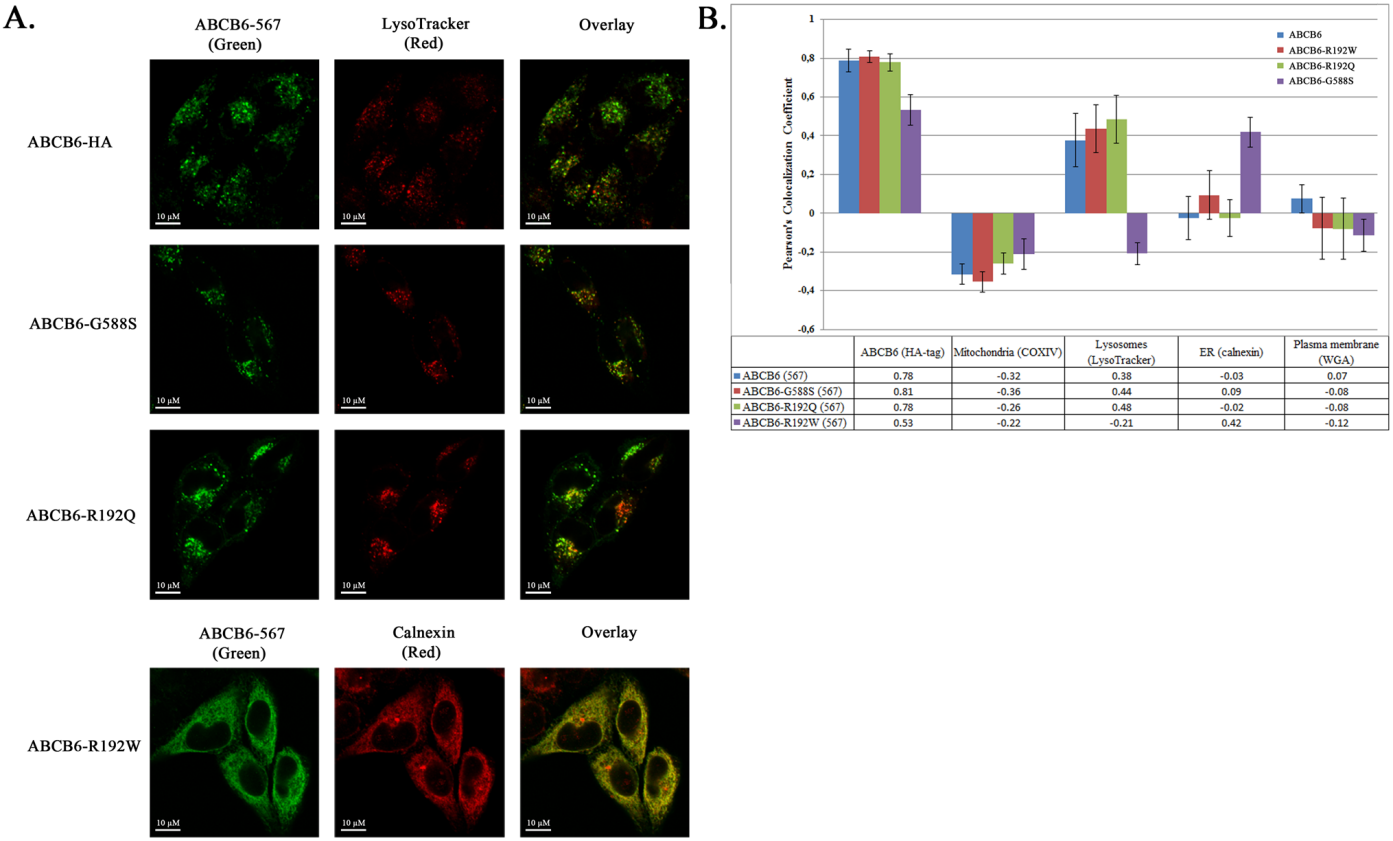
Determination of the subcellular localization of ABCB6 variants. A. Double immunofluorescence labeling and laser-scanning confocal microscopy images of HeLa cells overexpressing ABCB6-HA, ABCB6-HA-G588S, ABCB6-HA-R192Q or ABCB6-HA-R192W, probed with the ABCB6-567 antibody (green) [9] in the context of LysoTracker (red) or Calnexin (red). B. Colocalization of ABCB6-variants with various intracellular markers. Bars represesent averaged Pearson’s coefficients +/− standard deviation for each subcellular marker from 5 random pictures. Blue: ABCB6-HA; red: ABCB6-HA-G588S; green: ABCB6-HA-R192Q; purple: ABCB6-HA-R192W.

Next, we wanted to further analyze the apparent deleterious effect of the mutations identified in low RBC ABCB6 expressors using Hela cells stably expressing the wild-type or the mutant ABCB6 proteins. Since the mutations did not compromise overall expression levels, we analyzed the localization of ABCB6 variants in intracellular compartments using confocal microscopy. While initial reports indicated localization of ABCB6 in the outer mitochondrial membrane [7], [9], more recent studies detected ABCB6 in the plasma membrane [9], the Golgi apparatus [10], and in organelles of the vesicular system [11], [12], [13], [14], [15]. In line with the reports demonstrating the endolysosomal localization of ABCB6, our HA-tagged ABCB6 construct appeared in the lysosomal compartment of Hela cells, and not in the mitochondria (Figure 4). As opposed to the distinct endolysosomal pattern of the wild-type protein, the R192W variant showed ER localization, supporting the conclusion that the R192W mutation results in the ER-retention of the protein [28]. Although both the R192Q and R192W mutations are predicted to be potentially damaging by the PolyPhen-2 algorithm [29], the R192Q mutation did not change the expression pattern of ABCB6 in Hela cells. Similarly, the intracellular expression pattern of the G588 S variant did not differ from that of wild-type ABCB6 (Figure 4A, B).

**Figure 5:**
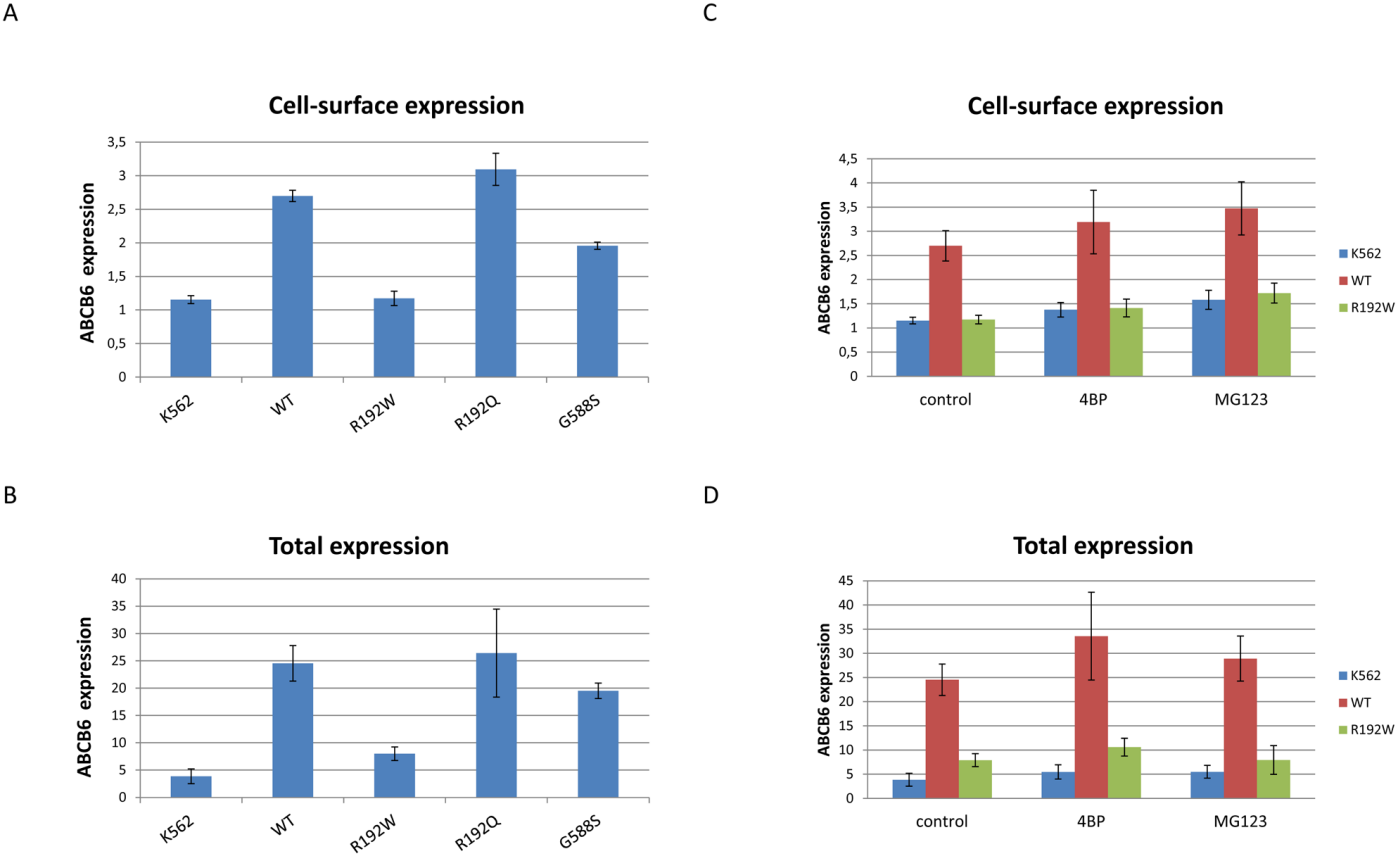
Detection of relative plasma membrane ABCB6 levels by flow cytometry. K562 cells were stably transduced with retroviral constructs encoding wild-type (WT), or mutated ABCB6 variants (R192W, R192Q, G588S). Cell surface (A, C) and total (B, D) ABCB6 expression was analyzed by flow cytometry using the monoclonal anti-Lan OSK43 antibody [16] in intact and permeabilized cells, respectively. The effect of pharmacological chaperoning and the inhibition of the proteasome (C, D) was studied by culturing the cells in the presence of 2 mM 4-PBA or 0.5 uM MG132, respectively. Bars represent average ABCB6 expression +/− standard deviation of at least 3 independent experiments.

Low RBC ABCB6 expression may be the result of a general folding defect, or may be linked to a yet unknown, erythrocyte-specific trafficking event. Red blood cells undergo significant membrane remodeling during the late stages of differentiation. During this process proteins residing in intracellular membrane compartments are redistributed to the plasma membrane of mature erythrocytes. Regulation of the intracellular trafficking and the molecular details of targeting of the ABCB6 protein are not known. In our experiments, endogenous ABCB6 is confined to the endolysosomal compartment in most cell lines, including the HeLa cells used in this study. We have also shown that C-terminal tags do not influence intracellular localization of ABCB6 [15] –still, there are numerous reasons why epithelial HeLa cells may not be an ideal model for membrane expression in erythrocytes. Therefore, we turned to K562 human erythroleukemia cells that are frequently used as a model system for erythroid cells [30]. Surface expression of the ABCB6 variants was monitored in intact cells, using the OSK43 antibody that recognizes an extracellular epitope (i.e., the Lan antigen) while global expression was monitored after permeabilizing the cells. Expression of the wild-type protein resulted in significant surface labeling, showing that 2–5% of the total protein population resides in the plasma membrane when ABCB6 is overexpressed in K562 cells. The R192Q and G588S mutations did not impede expression levels. The R192W variant did not reach the plasma membrane of K562 cells, in line with its ER retention in HeLa cells (Figure 5A, B). Disease-causing mutations of medically relevant ABC proteins (such as ABCC7-CFTR and ABCC6) often result in deficient folding, altered trafficking and a consequent degradation mediated by the proteasome [31], [32]. Treatment of K562 cells with the chemical chaperone 4-phenylbutyrate (4-PBA) or the proteasome inhibitor MG132 did not restore the expression of the R192W-ABCB6 variant, indicating that the folding defect could not be overcome (Figure 5C, D).

**Figure 6:**
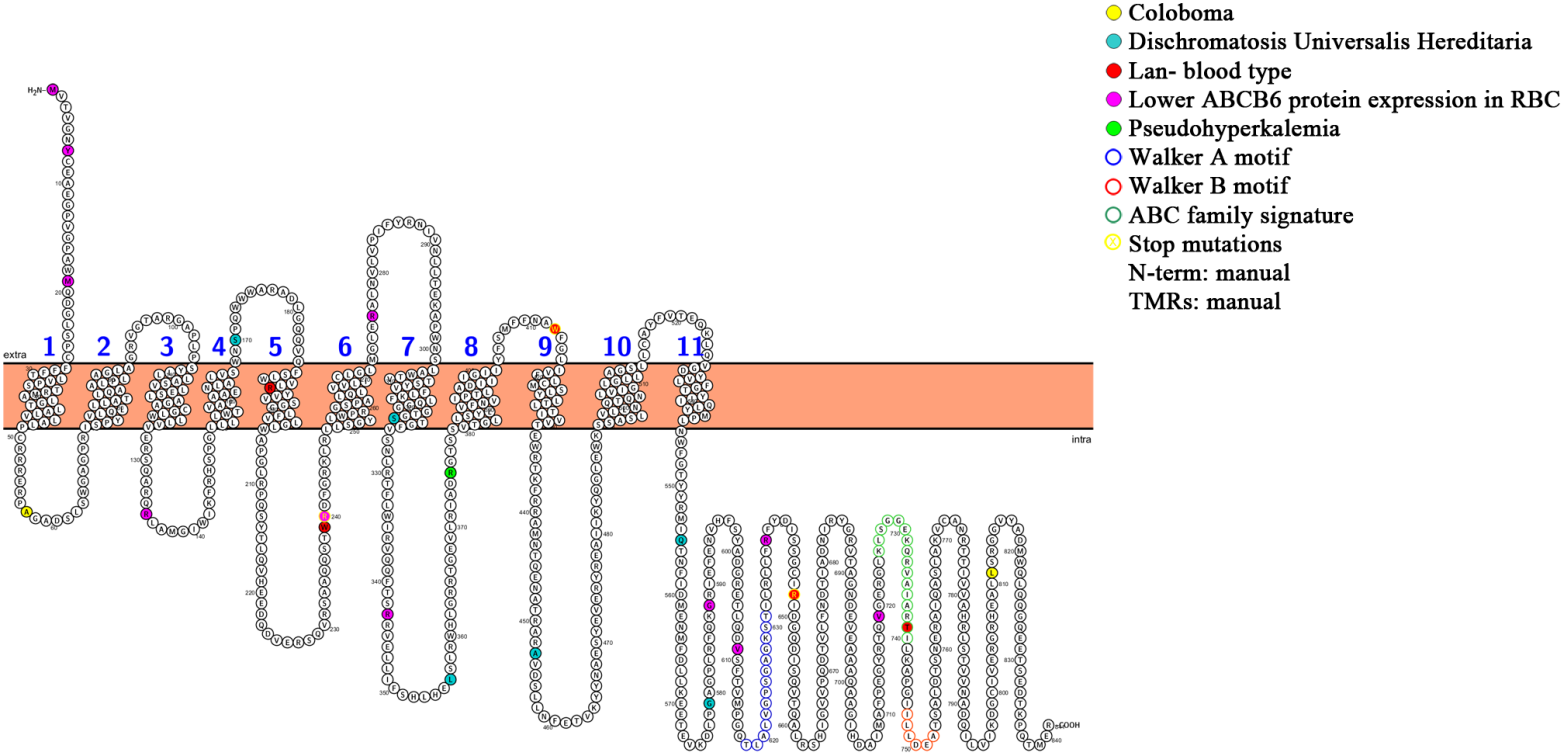
Topology model of ABC6 showing mutations and SNPs [39], [40].

The function of ABCB6 in red blood cells is unknown. The medical relevance of RBC ABCB6 expression is based on its association with severe haemolytic transfusion reactions or the mild haemolytic disease of the foetus and newborn. The Lan-negative blood type is believed to be very rare (1∶20000 random individuals are Lan-negative) [17], and is usually identified through the presence of anti-Lan antibodies stimulated by transfusion or pregnancy [33]. In this study we use a quantitative FACs assay and immunoblotting to associate genetic variations with reduced ABCB6 expression in red blood cells. Since weak expression of the ABCB6 protein may be sufficient to suppress the production of anti-Lan antibodies, further serological tests, based on the reactivity of anti-Lan containing sera, are needed to confirm the association of these mutations with Lan-negativity or the Lan-weak phenotype [34]. The Lan-phenotype is not screened routinely – in view of the genetic heterogeneity and the variable expression levels, genetic and serologic screening of the population is challenging and may be unrealistic [17]. To date, 34 alleles have been described in association with reduced ABCB6 expression in erythrocytes (a complete inventory is shown in Figure 6 and Table 1). Currently, there is no information available on the distribution of ABCB6 expression levels across the general population, healthy subpopulations, and disease-diagnosed individuals. Screening of our cohort revealed a surprisingly high frequency of mutations associated with reduced ABCB6 expression. To obtain carrier frequency data, we screened remaining individuals from the discovery cohort (n = 43) and a control cohort of 184 Hungarian blood donors for the presence of R192Q and G588 S mutations. Further individuals carrying the R192Q allele were not identified (suggesting an allele frequency (AF) of 0.2±0.2% (1/462)). The c.1762G>A, p.Gly588Ser mutation was present in 3 additional unrelated individuals, indicating an AF of 1.1±0.5% (5/462).

**Table 1:** Molecular genetics of the *ABCB6* gene.

NUCLEOTIDECHANGE	AMINO ACIDCHANGE	rs#	AF (%)(NCBI/this study)	EFFECT OFTHE SNP	SOURCE
1A>C				Lan (−) blood type	[41]
55A>T	M19L			Lan (−) blood type	[41]
85_87del	F29del			Lan (−) blood type	[28]
197_198insG	A66fs			Lan (−) blood type	[16]
296_301insG	A101fs			Lan (−) blood type	[42]
376delG	V126fs	rs377591749	N.D.	Lan (−) blood type	[17]
459del	L154fs			Lan (−) blood type	[42]
574C>T	R192W	rs149202834	0.09/0.86	Lan (−) blood type	[17] [28] this study
717G>A	Q239X	rs148458820	0.05	Lan (−) blood type	[16]
718C>T	R240X			Lan (−) blood type	[42]
827G>A	R276Q	rs200125320	N.D./0.27	Lan (−) blood type	[41] this study
881_884del	T294fs			Lan (−) blood type	[42]
953_956del	G318fs			Lan (−) blood type	[16]
971−1G>A	splice variant			Lan (−) blood type	[41]
1236G>A	W412X			Lan (−) blood type	[17]
1533_1543dup	L515fs			Lan (−) blood type	[16]
1558_1559insT	V520fs			Lan (−) blood type	[17]
1617delG	G539del			Lan (−) blood type	[42]
1690_1691del	M564fs			Lan (−) blood type	[16]
1709_1710del	Q570fs			Lan (−) blood type	[16]
1825G>A	V609M	rs374541748	N.D.	Lan (−) blood type	[41]
1867delins	G623fs			Lan (−) blood type	[16]
1912C>T	R638C			Lan (−) blood type	[41]
1942C>T	R648X	rs376664522	N.D.	Lan (−) blood type	[16]
1985_1986del	L662fs			Lan (−) blood type	[16] [17]
2155C>T	Q719X			Lan (−) blood type	[41]
2256+2T>G	splice variant			Lan (−) blood type	[16]
2351+1G>A	splice variant	rs150574070	N.D.	Lan (−) blood type	[41]
575G>A	R192Q	rs150221689	0.14/0.51	lower ABCB6 expression	this study
826C>T	R276W	rs57467915	0.6/1.18	lower ABCB6 expression	[17] [28] this study
1028G>A	R343Q	rs60322991	3.99	lower ABCB6 expression	[17]
1762G>A	G588S	rs145526996	0.23/0.98	lower ABCB6 expression	[17] [28] this study
1766G>A	R589H	rs483352876	N.D./0.04		this study
2216G>A	R739H	rs192931087	0.05	lower ABCB6 expression	[17]
1578+1G>A	splice variant		N.D./0.04	lower ABCB6 expression	this study
169G>A	A57T			coloboma	[19]
2431C>G	L811V			coloboma	[19]
508A>G	S170G			DUH	[21]
964A>C	S322L			DUH	[43]
1067T>C	L356P			DUH	[21]
1358C>T	A453V	rs373246472	N.D.	DUH	[43]
459delC				DUH	[44]
1663C>A	G555K			DUH	[44]
1736G>A	G579E			DUH	[21]
1123C>T	R375W			pseudohyperkalemia	[20]
1124G>A	R375Q			pseudohyperkalemia	[20]

Abbreviations: DUH: dyschromatosis universalis hereditaria; del: deletion; dup: duplication; fs: frameshift; ins: insertion; N.D.:not determined; rs: RefSNP.

In order to corroborate this finding, we analyzed a library of genetic material from 1039 healthy Polish individuals. This analysis (based on High Resolution Melting (HRM) screening followed by detailed sequencing analysis of variant individuals) also led to identification of a surprisingly high number of probands carrying mutations at the studied alleles (Table 1). At the R192 position, our analysis established the following frequencies: for the c.574C>T variant (p.R192W, rs149202834), we found 20 heterozygous and 1 homozygous individual, with an AF of 1.06±0.22%; for the c.575G>A variant (p.R192Q, rs150221689), we found 12 heterozygous individuals, with an AF of 0.58±0.17% (these polymorphisms are not allelomorphic as they concern separate, adjacent nucleotides within codon 192). At the codon 276, we also identified two non-allelomorphic variants: the c.826C>T (p.R276W, rs57467915) with 28 heterozygous and 1 homozygous individual, for an AF of 1.40±0.26%; and the c.827G>A (p.R276Q, rs200125320) with 7 heterozygous individuals, with an AF of 0.34±0.13%. Screening of the G588 position yielded 20 heterozygous individuals with the c.1762G>A (p.G588S, rs145526996) polymorphic variant, for an AF of 0.96±0.21%. In the same screen, we also identified an individual with a divergent HRM curve revealing a heterozygous carrier of a novel, hitherto unreported polymorphic variant in an adjacent codon: c.1766G>A (p.R589H), which we registered as rs483352876. No carriers of the splice site mutation (IVS9+1G>A) allele were identified in the Polish cohort. Interestingly, all 7 heterozygous carriers of the R276Q polymorphism also carried the G588S polymorphism (presumably on the same chromosome), suggesting a one-sided linkage between the two variants in the Polish population, perhaps due to a founder effect. The large size of the Polish cohort allowed us to confirm with relatively high certainty the high allele frequencies of the variants linked to weak or no expression of ABCB6 on RBCs.

Protein levels are heritable molecular phenotypes that exhibit considerable variation between individuals, populations and sexes [35]. Difference in gene expression levels is thought to be a major determinant of phenotypic variation. However, correlation between protein and mRNA levels is generally modest; variation in messenger RNA expression levels is not a perfect surrogate especially for membrane protein expression because the latter is influenced by an array of post-transcriptional regulatory mechanisms. Population proteomics is the protein-level equivalent to population genomics, where individuals are interrogated with the aim of cataloging common genetic variants and determining how they are distributed among people, within populations, and among populations in different parts of the world [36]. The quantitative analysis of the RBC membrane proteins may provide a new biomarker platform for this type of analysis. The red cell plasma membrane contains more than 200 proteins, mostly with unknown function in the mature erythrocyte, but with key physiological and/or pathological roles in other cell types [37], [38]. Interestingly, the RBC membrane protein levels in many cases reflect both the genetic alterations and the cellular regulation of the expression of these proteins [22], [38]. In the present work we show that the analysis of the ABCB6 expression in the RBC membrane allows the combined estimation of the expression and trafficking of this protein to the plasma membrane, as well as the discovery of heritable mutations related to the Lan-negative phenotype.

## Supporting Information

Figure S1 **RBC ABCB6 expression in 47 individuals.**
(TIF)
