# Supplementary figures and images for: Screening the Expression of ABCB6 in Erythrocytes Reveals an Unexpectedly High Frequency of Lan Mutations in Healthy Individuals
Source: PLoS One. 2014 Oct 31;9(10):e111590. doi: 10.1371/journal.pone.0111590 (PMC4216114; doi:10.1371/journal.pone.0111590)

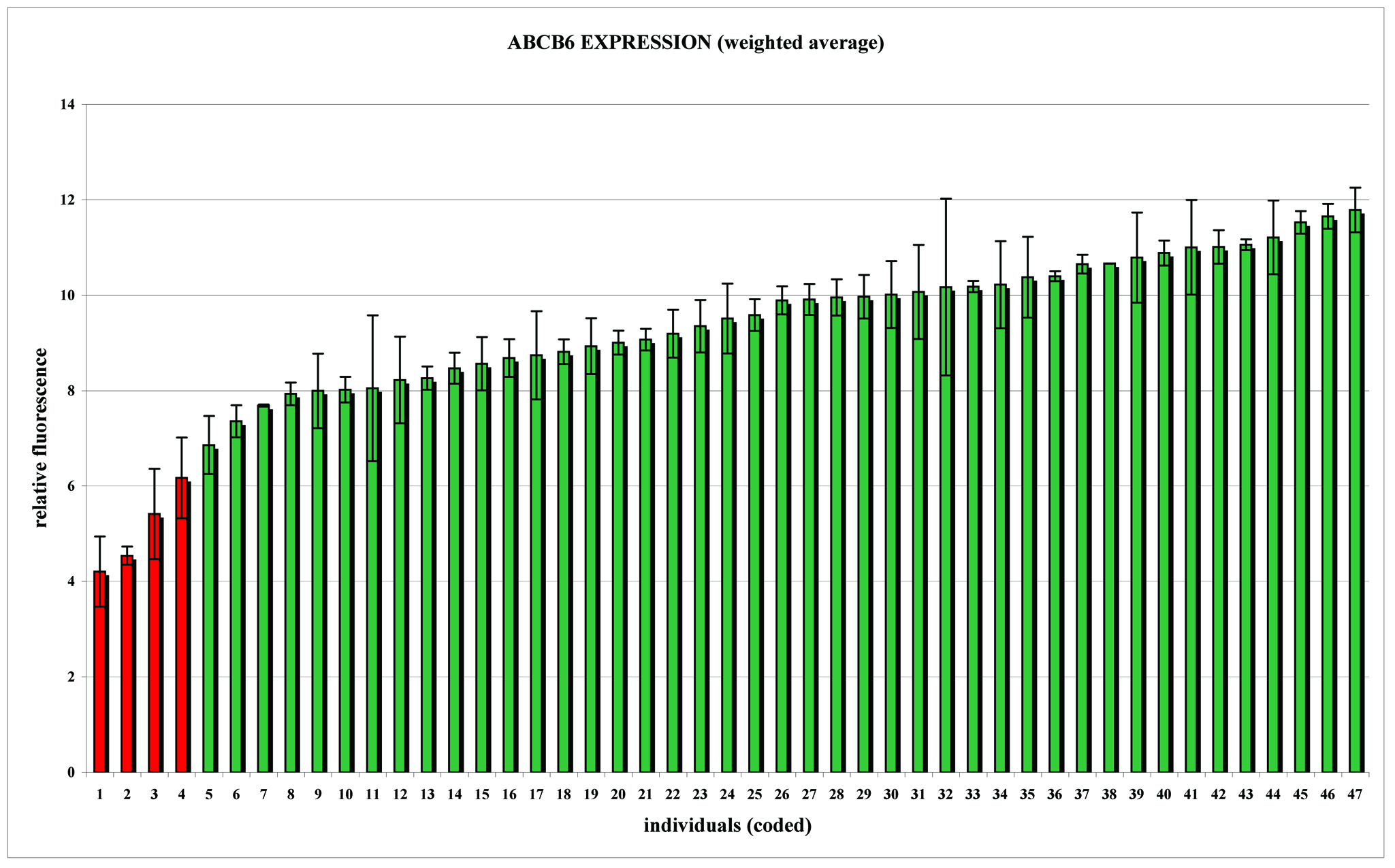

Supplement: Figure S1 — RBC ABCB6 expression in 47 individuals. (TIF) [file pone.0111590.s001.tif]
